# Cohort Differences in Older Adults’ Marital Status, Transitions, and Social and Mental Health

**DOI:** 10.1093/geroni/igaf122.3064

**Published:** 2025-12-31

**Authors:** Nell Compernolle, Ruoqi Zheng

**Affiliations:** NORC at the University of Chicago, Chicago, Illinois, United States; National Opinion Research Center at The University of Chicago, Chicago, Illinois, United States

## Abstract

**Background and Objectives:**

Long-examined links between marriage and health emphasize marital status and physical dimensions of health. This study expands current conversations by considering vastly under-studied never-married older adults, marital transitions on social and mental health dimensions, and variation across cohorts.

**Methods:**

We analyzed four rounds of data from the National Social Life, Health and Aging Project (NSHAP) spanning 2005 to 2021. A series of linear regression models were used to examine associations between marital status (currently, formerly, never married), transitions (in and out of marriage), and dimensions of health (loneliness, happiness, depression), and moderation by cohort (the Silent Generation, Baby Boomers), respectively.

**Results:**

Our findings suggest marriage benefits, but only for certain dimensions: transitions mattered for loneliness and, to some extent depression, whereas status mattered for happiness. Interestingly, Baby Boomers’ happiness is more strongly associated both with their marital status and transitions than it is for the Silent Generation. Next, never-married older adults comprise a population with unique experiences from other singles: they do not differ from their married peers in terms of happiness or depression; they are lonelier but not as lonely as those formerly married. Finally, never-married Baby Boomers were less depressed than those never-married in the Silent Generation.

**Discussion:**

Explicit consideration of never-married older adults and cohort differences provide novel insights into long-studied marriage-health links. Specifically, the social context—marriage norms—in which older adults entered and experience marriage and any subsequent transitions continue to shape their social and mental health in later life.

